# Supplementary material for: Effects of high-intensity interval training versus moderate-intensity continuous training on cardiorespiratory and exercise capacity in patients with coronary artery disease: A systematic review and meta-analysis
Source: PLoS One. 2025 Feb 20;20(2):e0314134. doi: 10.1371/journal.pone.0314134 (PMC11841918; doi:10.1371/journal.pone.0314134)
Supplement: S2 File — (DOCX) [file pone.0314134.s002.docx]

| **Supplemental File 2** |  |
| --- | --- |
| **PUBMED** |  |
| Mesh | **Coronary Artery Disease** |
| Entry  Terms | Artery Disease, Coronary  Artery Diseases, Coronary  Coronary Artery Diseases  Coronary Arteriosclerosis  Arterioscleroses, Coronary  Coronary Arterioscleroses  Arteriosclerosis, Coronary  Atherosclerosis, Coronary  Atheroscleroses, Coronary  Coronary Atheroscleroses  Coronary Atherosclerosis  Left Main Coronary Artery Disease  Left Main Coronary Disease  Left Main Disease  Left Main Diseases |
| #1 | ****((((((((((((((("Coronary Artery Disease"[Mesh]) OR (Artery Disease, Coronary[Title/Abstract])) OR (Artery Diseases, Coronary[Title/Abstract])) OR (Coronary Artery Diseases[Title/Abstract])) OR (Coronary Arteriosclerosis[Title/Abstract])) OR (Arterioscleroses, Coronary[Title/Abstract])) OR (Coronary Arterioscleroses[Title/Abstract])) OR (Arteriosclerosis, Coronary[Title/Abstract])) OR (Atherosclerosis, Coronary[Title/Abstract])) OR (Atheroscleroses, Coronary[Title/Abstract])) OR (Coronary Atheroscleroses[Title/Abstract])) OR (Coronary Atherosclerosis[Title/Abstract])) OR (Left Main Coronary Artery Disease[Title/Abstract])) OR (Left Main Coronary Disease[Title/Abstract])) OR (Left Main Disease[Title/Abstract])) OR (Left Main Diseases[Title/Abstract])**** |
| Mesh | **Myocardial Ischemia** |
| Entry  Terms | Heart Disease, Ischemic  Disease, Ischemic Heart  Diseases, Ischemic Heart  Heart Diseases, Ischemic  Ischemic Heart Diseases  Ischemia, Myocardial  Ischemias, Myocardial  Myocardial Ischemias  Ischemic Heart Disease |
| #2 | ****((((((((("Myocardial Ischemia"[Mesh]) OR (Heart Disease, Ischemic[Title/Abstract])) OR (Disease, Ischemic Heart[Title/Abstract])) OR (Diseases, Ischemic Heart[Title/Abstract])) OR (Heart Diseases, Ischemic[Title/Abstract])) OR (Ischemic Heart Diseases[Title/Abstract])) OR (Ischemia, Myocardial[Title/Abstract])) OR (Ischemias, Myocardial[Title/Abstract])) OR (Myocardial Ischemias[Title/Abstract])) OR (Ischemic Heart Disease[Title/Abstract])**** |
| Mesh | **Acute Coronary Syndrome** |
| Entry  Terms | Acute Coronary Syndromes  Coronary Syndrome, Acute  Coronary Syndromes, Acute  Syndrome, Acute Coronary  Syndromes, Acute Coronary |
| #3 | ****((((("Acute Coronary Syndrome"[Mesh]) OR (Acute Coronary Syndromes[Title/Abstract])) OR (Coronary Syndrome, Acute[Title/Abstract])) OR (Coronary Syndromes, Acute[Title/Abstract])) OR (Syndrome, Acute Coronary[Title/Abstract])) OR (Syndromes, Acute Coronary[Title/Abstract])**** |
| Mesh | **percutaneous coronary intervention** |
| Entry  Terms | Coronary Intervention, Percutaneous  Coronary Interventions, Percutaneous  Intervention, Percutaneous Coronary  Interventions, Percutaneous Coronary  Percutaneous Coronary Interventions  Percutaneous Coronary Revascularization  Coronary Revascularization, Percutaneous  Coronary Revascularizations, Percutaneous  Percutaneous Coronary Revascularizations  Revascularization, Percutaneous Coronary  Revascularizations, Percutaneous Coronary |
| #4 | ****((((((((((("Percutaneous Coronary Intervention"[Mesh]) OR (Coronary Intervention, Percutaneous[Title/Abstract])) OR (Coronary Interventions, Percutaneous[Title/Abstract])) OR (Intervention, Percutaneous Coronary[Title/Abstract])) OR (Interventions, Percutaneous Coronary[Title/Abstract])) OR (Percutaneous Coronary Interventions[Title/Abstract])) OR (Percutaneous Coronary Revascularization[Title/Abstract])) OR (Coronary Revascularization, Percutaneous[Title/Abstract])) OR (Coronary Revascularizations, Percutaneous[Title/Abstract])) OR (Percutaneous Coronary Revascularizations[Title/Abstract])) OR (Revascularization, Percutaneous Coronary[Title/Abstract])) OR (Revascularizations, Percutaneous Coronary[Title/Abstract])**** |
| Mesh | **Myocardial Infarction** |
| Entry  Terms | Infarction, Myocardial  Infarctions, Myocardial  Myocardial Infarctions  Heart Attack  Heart Attacks  Myocardial Infarct  Infarct, Myocardial  Infarcts, Myocardial  Myocardial Infarcts  Cardiovascular Stroke  Cardiovascular Strokes  Stroke, Cardiovascular  Strokes, Cardiovascular |
| #5 | ****((((((((((((("Myocardial Infarction"[Mesh]) OR (Infarction, Myocardial[Title/Abstract])) OR (Infarctions, Myocardial[Title/Abstract])) OR (Myocardial Infarctions[Title/Abstract])) OR (Heart Attack[Title/Abstract])) OR (Heart Attacks[Title/Abstract])) OR (Myocardial Infarct[Title/Abstract])) OR (Infarct, Myocardial[Title/Abstract])) OR (Infarcts, Myocardial[Title/Abstract])) OR (Myocardial Infarcts[Title/Abstract])) OR (Cardiovascular Stroke[Title/Abstract])) OR (Cardiovascular Strokes[Title/Abstract])) OR (Stroke, Cardiovascular[Title/Abstract])) OR (Strokes, Cardiovascular[Title/Abstract])**** |
| Mesh | **High Intensity Interval Training** |
| Entry  Terms | High-Intensity Interval Trainings  Interval Training, High-Intensity  Interval Trainings, High-Intensity  Training, High-Intensity Interval  Trainings, High-Intensity Interval  High-Intensity Intermittent Exercise  Exercise, High-Intensity Intermittent  Exercises, High-Intensity Intermittent  High-Intensity Intermittent Exercises  Sprint Interval Training  Sprint Interval Trainings |
| #6 | ****(((((((((((("High-Intensity Interval Training"[Mesh]) OR (High Intensity Interval Training[Title/Abstract])) OR (High-Intensity Interval Trainings[Title/Abstract])) OR (Interval Training, High-Intensity[Title/Abstract])) OR (Interval Trainings, High-Intensity[Title/Abstract])) OR (Training, High-Intensity Interval[Title/Abstract])) OR (Trainings, High-Intensity Interval[Title/Abstract])) OR (High-Intensity Intermittent Exercise[Title/Abstract])) OR (Exercise, High-Intensity Intermittent[Title/Abstract])) OR (Exercises, High-Intensity Intermittent[Title/Abstract])) OR (High-Intensity Intermittent Exercises[Title/Abstract])) OR (Sprint Interval Training[Title/Abstract])) OR (Sprint Interval Trainings[Title/Abstract])**** |
| #7 | 1 or 2 or 3 or 4 or 5 |
| #8 | #7 AND #6 |

|  |  |
| --- | --- |
| **Web of science** |  |
| #1 | **(((((((((((((((TS=(Coronary Artery Disease)) OR TS=(Artery Disease, Coronary)) OR TS=(Artery Diseases, Coronary)) OR TS=(Coronary Artery Diseases)) OR TS=(Coronary Arteriosclerosis)) OR TS=(Arterioscleroses, Coronary)) OR TS=(Coronary Arterioscleroses)) OR TS=(Arteriosclerosis, Coronary)) OR TS=(Atherosclerosis, Coronary)) OR TS=(Atheroscleroses, Coronary)) OR TS=(Coronary Atheroscleroses)) OR TS=(Coronary Atherosclerosis)) OR TS=(Left Main Coronary Artery Disease)) OR TS=(Left Main Coronary Disease)) OR TS=(Left Main Disease)) OR TS=(Left Main Diseases)** |
| #2 | **(((((((((TS=(Myocardial Ischemia)) OR TS=(Heart Disease, Ischemic)) OR TS=(Disease, Ischemic Heart)) OR TS=(Diseases, Ischemic Heart)) OR TS=(Heart Diseases, Ischemic)) OR TS=(Ischemic Heart Diseases)) OR TS=(Ischemia, Myocardial)) OR TS=(Ischemias, Myocardial)) OR TS=(Myocardial Ischemias)) OR TS=(Ischemic Heart Disease)** |
| #3 | **(((((TS=(Acute Coronary Syndrome)) OR TS=(Acute Coronary Syndromes)) OR TS=(Coronary Syndrome, Acute)) OR TS=(Coronary Syndromes, Acute)) OR TS=(Syndrome, Acute Coronary)) OR TS=(Syndromes, Acute Coronary)** |
| #4 | **(((((((((((TS=(percutaneous coronary intervention)) OR TS=(Coronary Intervention, Percutaneous)) OR TS=(Coronary Interventions, Percutaneous)) OR TS=(Intervention, Percutaneous Coronary)) OR TS=(Interventions, Percutaneous Coronary)) OR TS=(Percutaneous Coronary Interventions)) OR TS=(Percutaneous Coronary Revascularization)) OR TS=(Coronary Revascularization, Percutaneous)) OR TS=(Coronary Revascularizations, Percutaneous)) OR TS=(Percutaneous Coronary Revascularizations)) OR TS=(Revascularization, Percutaneous Coronary)) OR TS=(Revascularizations, Percutaneous Coronary)** |
| #5 | **(((((((((((((TS=(Myocardial Infarction)) OR TS=(Infarction, Myocardial)) OR TS=(Infarctions, Myocardial)) OR TS=(Myocardial Infarctions)) OR TS=(Heart Attack)) OR TS=(Heart Attacks)) OR TS=(Myocardial Infarct)) OR TS=(Infarct, Myocardial)) OR TS=(Infarcts, Myocardial)) OR TS=(Myocardial Infarcts)) OR TS=(Cardiovascular Stroke)) OR TS=(Cardiovascular Strokes)) OR TS=(Stroke, Cardiovascular)) OR TS=(Strokes, Cardiovascular)** |
| #6 | **(((((((((((TS=(High Intensity Interval Training)) OR TS=(High-Intensity Interval Trainings)) OR TS=(Interval Training, High-Intensity)) OR TS=(Interval Trainings, High-Intensity)) OR TS=(Training, High-Intensity Interval)) OR TS=(Trainings, High-Intensity Interval)) OR TS=(High-Intensity Intermittent Exercise)) OR TS=(Exercise, High-Intensity Intermittent)) OR TS=(Exercises, High-Intensity Intermittent)) OR TS=(High-Intensity Intermittent Exercises)) OR TS=(Sprint Interval Training)) OR TS=(Sprint Interval Trainings)** |
| #7 | #1 or #2 or #3 or #4 or #5 |
| #8 | #7 AND #6 |

|  |  |
| --- | --- |
| **Embase** |  |
| #1 | 'coronary disease'/exp OR 'coronary disease' OR 'multivessel coronary artery disease'/exp OR 'multivessel coronary artery disease' OR 'coronary artery disease'/exp OR 'coronary artery disease' |
| #2 | 'acute heart muscle ischaemia'/exp OR 'acute heart muscle ischaemia' OR 'acute heart muscle ischemia'/exp OR 'acute heart muscle ischemia' OR 'cardiac ischaemia'/exp OR 'cardiac ischaemia' OR 'cardiac ischemia'/exp OR 'cardiac ischemia' OR 'cardiac muscle ischaemia'/exp OR 'cardiac muscle ischaemia' OR 'cardiac muscle ischemia'/exp OR 'cardiac muscle ischemia' OR 'coronary artery ischaemia'/exp OR 'coronary artery ischaemia' OR 'coronary artery ischemia'/exp OR 'coronary artery ischemia' OR 'coronary ischaemia'/exp OR 'coronary ischaemia' OR 'coronary ischemia'/exp OR 'coronary ischemia' OR 'coronary syndrome'/exp OR 'coronary syndrome' OR 'heart anoxia'/exp OR 'heart anoxia' OR 'heart hypoxia'/exp OR 'heart hypoxia' OR 'heart ischaemia'/exp OR 'heart ischaemia' OR 'heart ischaemic arrest'/exp OR 'heart ischaemic arrest' OR 'heart ischaemic attack'/exp OR 'heart ischaemic attack' OR 'heart ischaemic time'/exp OR 'heart ischaemic time' OR 'heart ischemia'/exp OR 'heart ischemia' OR 'heart ischemic arrest'/exp OR 'heart ischemic arrest' OR 'heart ischemic attack'/exp OR 'heart ischemic attack' OR 'heart ischemic time'/exp OR 'heart ischemic time' OR 'heart muscle hypoxia'/exp OR 'heart muscle hypoxia' OR 'heart muscle ischaemia'/exp OR 'heart muscle ischaemia' OR 'heart muscle ischaemia, subepicardial'/exp OR 'heart muscle ischaemia, subepicardial' OR 'heart muscle ischemia, subepicardial'/exp OR 'heart muscle ischemia, subepicardial' OR 'heart transient ischaemic attack'/exp OR 'heart transient ischaemic attack' OR 'heart transient ischemic attack'/exp OR 'heart transient ischemic attack' OR 'hypoxia, heart'/exp OR 'hypoxia, heart' OR 'hypoxic heart'/exp OR 'hypoxic heart' OR 'ischaemic heart'/exp OR 'ischaemic heart' OR 'ischaemic heart arrest'/exp OR 'ischaemic heart arrest' OR 'ischaemic myocardium'/exp OR 'ischaemic myocardium' OR 'ischemic heart'/exp OR 'ischemic heart' OR 'ischemic heart arrest'/exp OR 'ischemic heart arrest' OR 'ischemic myocardium'/exp OR 'ischemic myocardium' OR 'myocardial anoxia'/exp OR 'myocardial anoxia' OR 'myocardial hypoxia'/exp OR 'myocardial hypoxia' OR 'myocardial ischaemia'/exp OR 'myocardial ischaemia' OR 'myocardial ischemia'/exp OR 'myocardial ischemia' OR 'myocardium hypoxia'/exp OR 'myocardium hypoxia' OR 'myocardium ischaemia'/exp OR 'myocardium ischaemia' OR 'myocardium ischemia'/exp OR 'myocardium ischemia' OR 'subendocardial ischaemia'/exp OR 'subendocardial ischaemia' OR 'subendocardial ischemia'/exp OR 'subendocardial ischemia' OR 'transient ischaemic attack, heart'/exp OR 'transient ischaemic attack, heart' OR 'transient ischemic attack, heart'/exp OR 'transient ischemic attack, heart' OR 'heart muscle ischemia'/exp OR 'heart muscle ischemia' |
| #3 | 'acute coronary syndromes'/exp OR 'acute coronary syndromes' OR 'acute coronary syndrome'/exp OR 'acute coronary syndrome' |
| #4 | 'percutaneous coronary intervention'/exp OR 'percutaneous coronary intervention' |
| #5 | 'cardiac infarct'/exp OR 'cardiac infarct' OR 'cardiac infarction'/exp OR 'cardiac infarction' OR 'cardial infarct'/exp OR 'cardial infarct' OR 'heart attack'/exp OR 'heart attack' OR 'heart infarct'/exp OR 'heart infarct' OR 'heart micro infarction'/exp OR 'heart micro infarction' OR 'heart muscle infarction'/exp OR 'heart muscle infarction' OR 'infarction, heart'/exp OR 'infarction, heart' OR 'myocardial infarct'/exp OR 'myocardial infarct' OR 'myocardial infarction'/exp OR 'myocardial infarction' OR 'myocardium infarct'/exp OR 'myocardium infarct' OR 'myocardium infarction'/exp OR 'myocardium infarction' OR 'premonitory infarction sign'/exp OR 'premonitory infarction sign' OR 'second heart attack'/exp OR 'second heart attack' OR 'subendocardial infarction'/exp OR 'subendocardial infarction' OR 'transmural cardiac infarction'/exp OR 'transmural cardiac infarction' OR 'transmural heart infarction'/exp OR 'transmural heart infarction' OR 'transmural infarction, heart'/exp OR 'transmural infarction, heart' OR 'heart infarction'/exp OR 'heart infarction' |
| #6 | 'high‐intensity interval training' OR 'high-intensity intermittent exercise'/exp OR 'high-intensity intermittent exercise' OR 'high-intensity intermittent training'/exp OR 'high-intensity intermittent training' OR 'high-intensity interval exercise'/exp OR 'high-intensity interval exercise' OR 'high-intensity interval training'/exp OR 'high-intensity interval training' OR 'hiie (exercise)'/exp OR 'hiie (exercise)' OR 'hiit'/exp OR 'hiit' OR 'intermittent high-intensity training'/exp OR 'intermittent high-intensity training' OR 'interval high-intensity training'/exp OR 'interval high-intensity training' OR 'high intensity interval training'/exp OR 'high intensity interval training' |
| #7 | #1 or #2 or #3 or #4 or #5 |
| #8 | #7 AND #6 |

|  |  |
| --- | --- |
| **[Cochrane Library](https://www.cochranelibrary.com/library)** |  |
| #1 | **Coronary Artery Disease** |
| #2 | (Coronary Artery Disease OR Arteriosclerosis, Coronary OR Coronary Arteriosclerosis OR Coronary Atherosclerosis OR Coronary Atheroscleroses OR Arterioscleroses, Coronary OR Coronary Arterioscleroses OR Atherosclerosis, Coronary OR Atheroscleroses, Coronary OR Coronary Artery Diseases OR Artery Diseases, Coronary OR Artery Disease, Coronary OR Left Main Coronary Disease OR Left Main Diseases OR Left Main Disease OR Left Main Coronary Artery Disease):ti,ab,kw |
| #3 | **#1 or #2** |
| #4 | **Myocardial Ischemia** |
| #5 | (Myocardial Ischemia OR Ischemia, Myocardial OR Ischemic Heart Diseases OR Ischemias, Myocardial OR Heart Diseases, Ischemic OR Myocardial Ischemias OR Heart Disease, Ischemic OR Ischemic Heart Disease OR Disease, Ischemic Heart OR Diseases, Ischemic Heart):ti,ab,kw |
| #6 | **#4 or #5** |
| #7 | **Acute Coronary Syndrome** |
| #8 | (Acute Coronary Syndrome OR Coronary Syndromes, Acute OR Acute Coronary Syndromes OR Syndrome, Acute Coronary OR Syndromes, Acute Coronary OR Coronary Syndrome, Acute):ti,ab,kw |
| #9 | **#7 or#8** |
| #10 | **percutaneous coronary intervention** |
| #11 | (percutaneous coronary intervention OR Percutaneous Coronary Revascularizations OR Revascularization, Percutaneous Coronary OR Coronary Revascularization, Percutaneous OR Coronary Intervention, Percutaneous OR Coronary Interventions, Percutaneous OR Revascularizations, Percutaneous Coronary OR Percutaneous Coronary Revascularization OR Coronary Revascularizations, Percutaneous OR Percutaneous Coronary Interventions OR Intervention, Percutaneous Coronary OR Interventions, Percutaneous Coronary):ti,ab,kw |
| #12 | **#10 or #11** |
| #13 | **[High-Intensity Interval Training](http://www.cochranelibrary.g.yyttgd.top/advanced-search/mesh?term=high%E2%80%90intensity+interval+training&qualifier=#0)** |
| #14 | (High-Intensity Interval Training OR Sprint Interval Training OR Sprint Interval Trainings OR Trainings, High-Intensity Interval OR Interval Trainings, High-Intensity OR Training, High-Intensity Interval OR High-Intensity Intermittent Exercises OR Interval Training, High-Intensity OR High-Intensity Interval Trainings OR Exercise, High-Intensity Intermittent OR High Intensity Interval Training OR Exercises, High-Intensity Intermittent OR High-Intensity Intermittent Exercise):ti,ab,kw |
| #15 | #13 or #14 |
| #16 | #3 or #6 or #9 or #12 |
| #17 | #16 and #15 |

|  |  |
| --- | --- |
| **CINAHL** |  |
| #1 | Coronary Artery Disease OR TI ( Artery Disease, Coronary or Artery Diseases, Coronary or Coronary Artery Diseases or CoronaryArteriosclerosis or Arterioscleroses, Coronary or Coronary Arterioscleroses or Arteriosclerosis, Coronary or Atherosclerosis, Coronary or Atheroscleroses, Coronary or Coronary Atheroscleroses or Coronary Atherosclerosis or Left Main Coronary Artery Disease or Left Main Coronary Disease or Left Main Disease or Left Main Diseases ) |
| #2 | Myocardial Ischemia OR TI ( Heart Disease, Ischemic or Disease, Ischemic Heart or Diseases, Ischemic Heart or Heart Diseases, Ischemic or Ischemic Heart Diseases or Ischemia, Myocardial or Ischemias, Myocardial or Myocardial Ischemias or Ischemic Heart Disease ) |
| #3 | Acute Coronary Syndrome OR TI ( Acute Coronary Syndromes or Coronary Syndrome, Acute or Coronary Syndromes, Acute or Syndrome, Acute Coronary or Syndromes, Acute Coronary ) |
| #4 | percutaneous coronary intervention OR TI ( Coronary Intervention, Percutaneous or Coronary Interventions, Percutaneous or Intervention, Percutaneous Coronary or Interventions, Percutaneous Coronary or Percutaneous Coronary Interventions or Percutaneous Coronary Revascularization or Coronary Revascularization, Percutaneous or Coronary Revascularizations, Percutaneous or Percutaneous Coronary Revascularizations or Revascularization, Percutaneous Coronary or Revascularizations, Percutaneous Coronary ) |
| #5 | Myocardial Infarction OR TI ( Infarction, Myocardial or Infarctions, Myocardial or Myocardial Infarctions or Heart Attack or Heart Attacks or Myocardial Infarct or Infarct, Myocardial or Infarcts, Myocardial or Myocardial Infarcts or Cardiovascular Stroke or Cardiovascular Strokes or Stroke, Cardiovascular or Strokes, Cardiovascular ) |
| #6 | high‐intensity interval training OR TI ( High Intensity Interval Training or High-Intensity Interval Trainings or cInterval Trainings, High-Intensity or Training, High-Intensity Interval or Trainings, High-Intensity Interval or High-Intensity Intermittent Exercise or Exercise, High-Intensity Intermittent or Exercises, High-Intensity Intermittent or High-Intensity Intermittent Exercises or Sprint Interval Training or Sprint Interval Trainings ) |
| #7 | #1 or #2 or #3 or #4 or #5 |
| #8 | #7 AND #6 |
