# Supplementary figures and images for: Effects of high-intensity interval training versus moderate-intensity continuous training on cardiorespiratory and exercise capacity in patients with coronary artery disease: A systematic review and meta-analysis
Source: PLoS One. 2025 Feb 20;20(2):e0314134. doi: 10.1371/journal.pone.0314134 (PMC11841918; doi:10.1371/journal.pone.0314134)

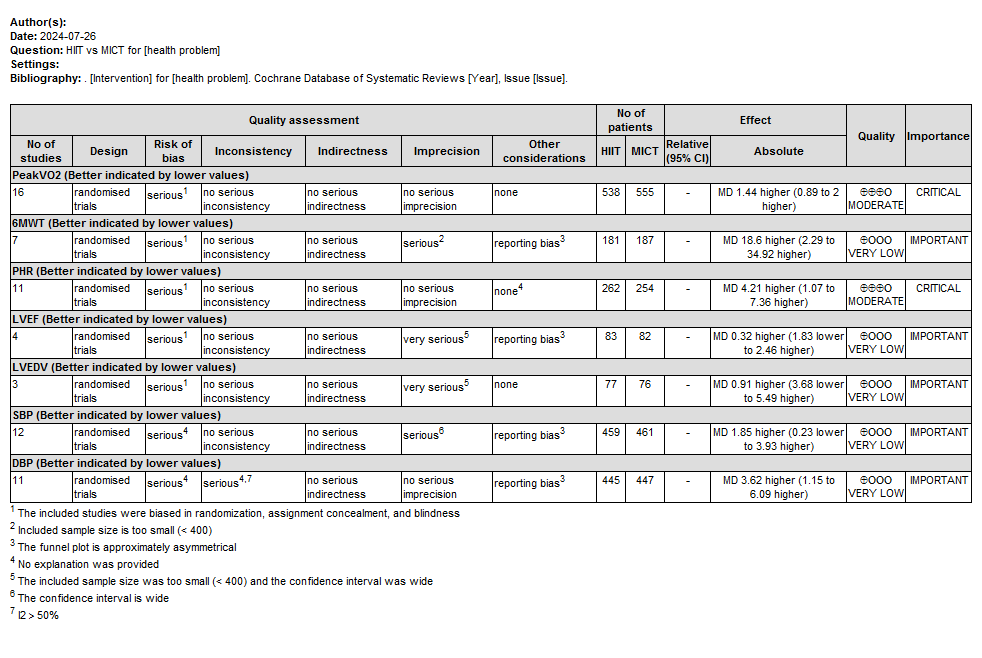

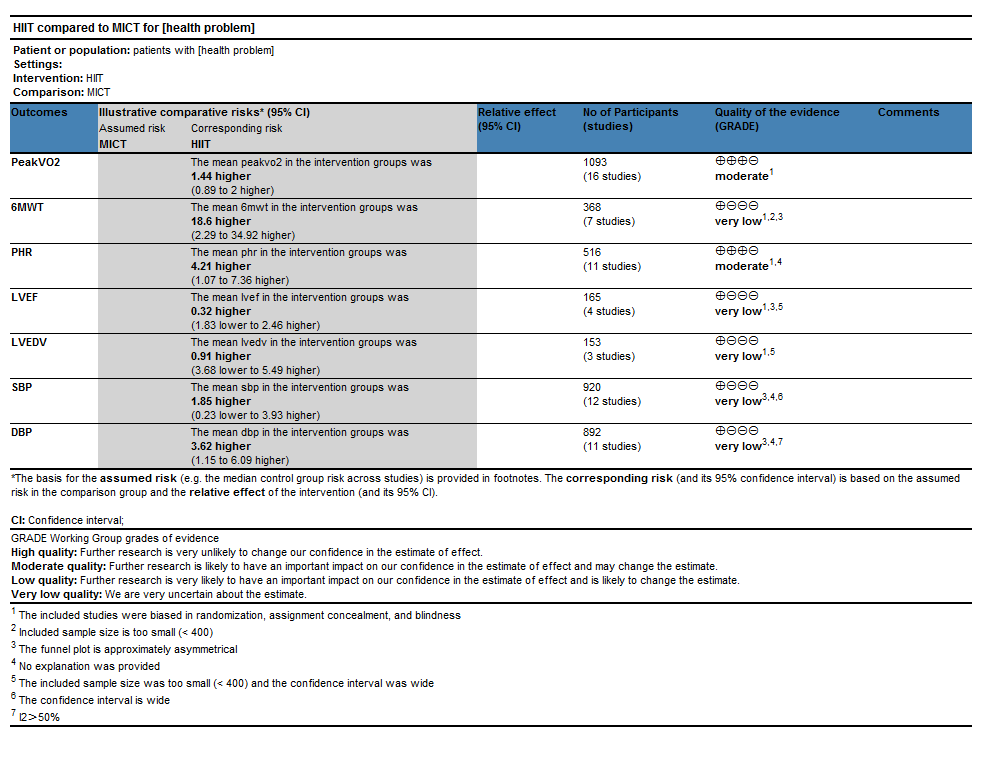

Supplement: S3 File — (DOCX) [file pone.0314134.s003.docx]

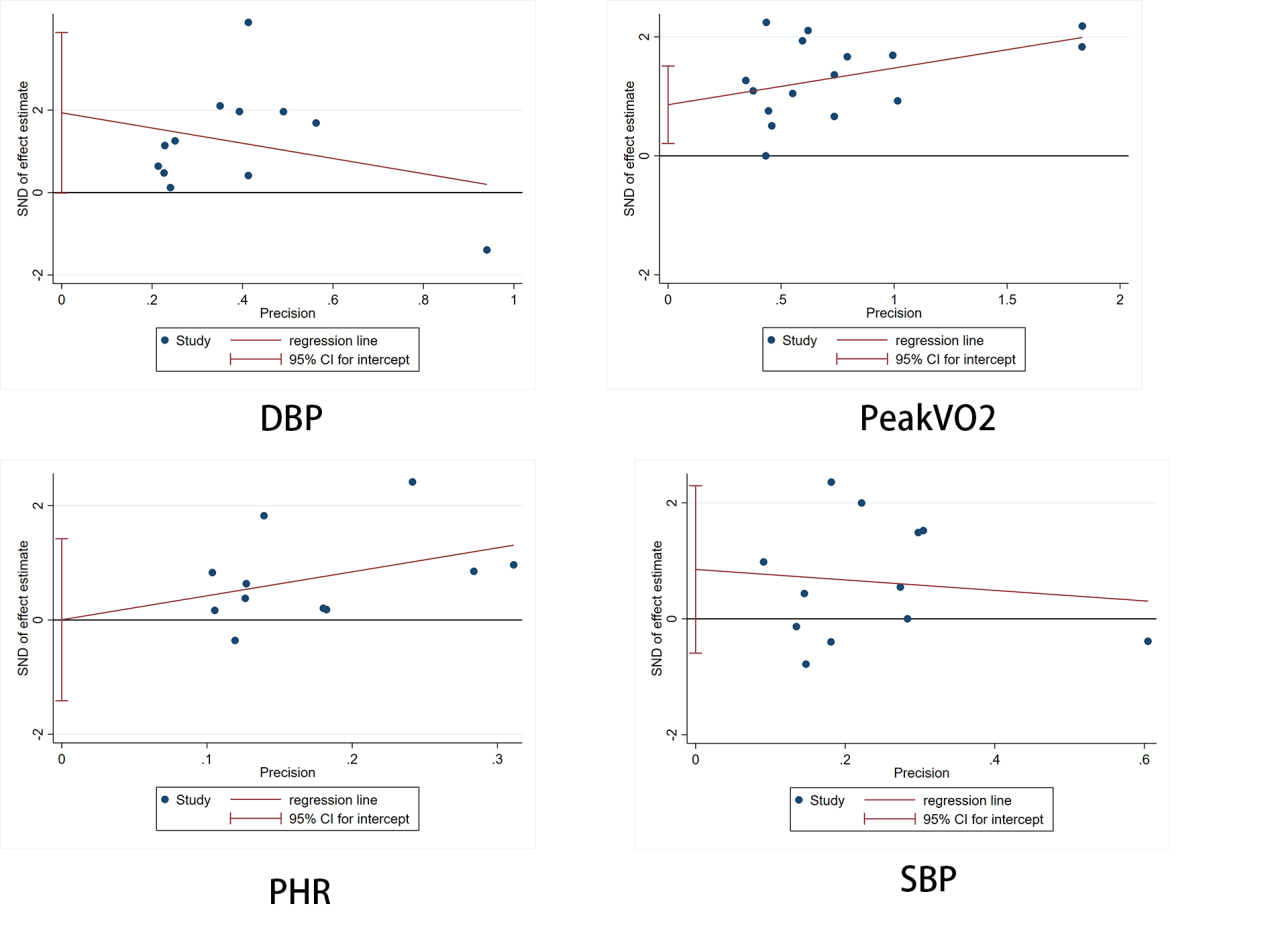

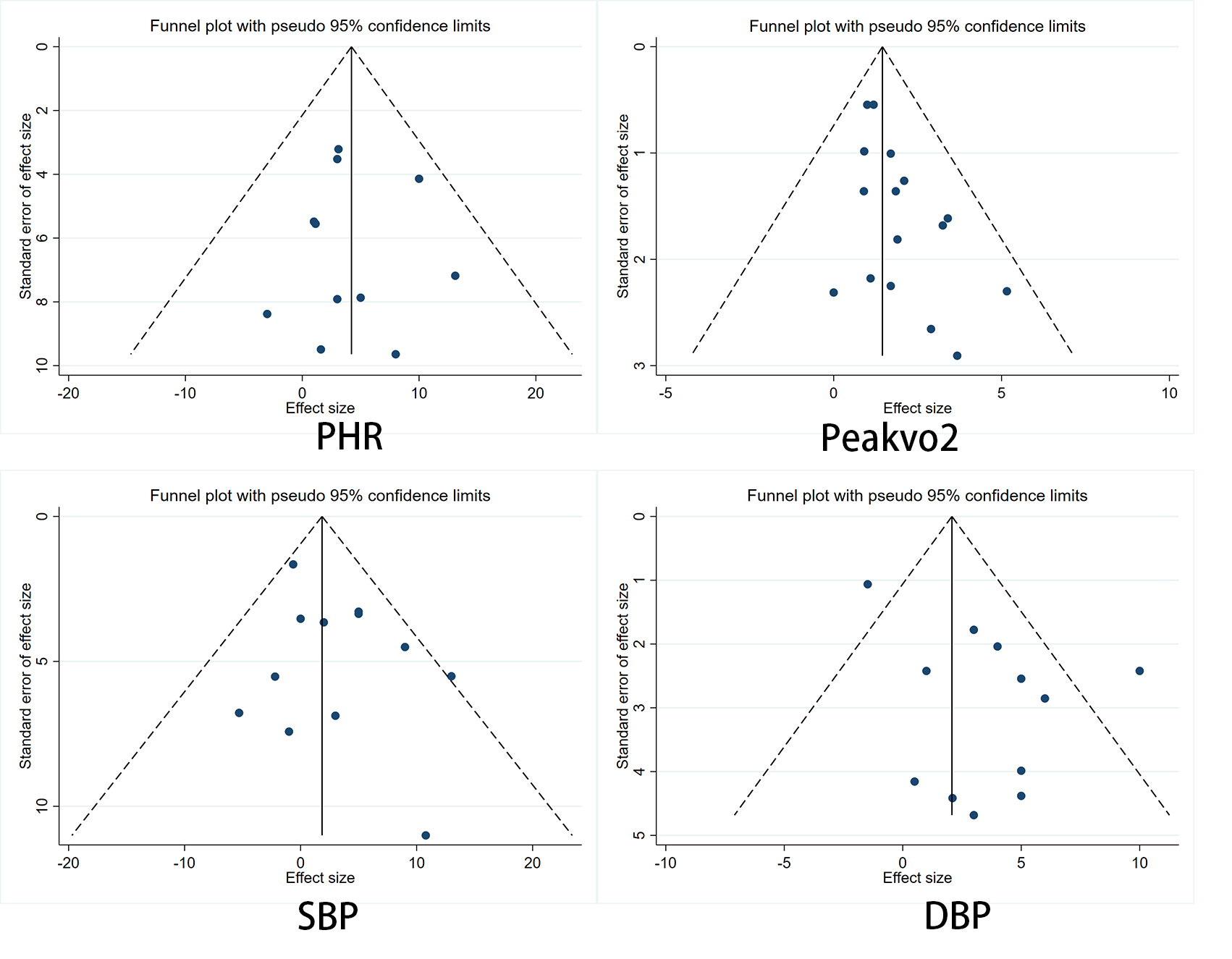

Supplement: S4 File — (DOCX) [file pone.0314134.s004.docx]
