# Supplementary material for: Effects of high-intensity interval training versus moderate-intensity continuous training on cardiorespiratory and exercise capacity in patients with coronary artery disease: A systematic review and meta-analysis
Source: PLoS One. 2025 Feb 20;20(2):e0314134. doi: 10.1371/journal.pone.0314134 (PMC11841918; doi:10.1371/journal.pone.0314134)
Supplement: Attachment — Submitted filename: Response Letter..docx [file pone.0314134.s006.docx]

**Response Letter**

Dear editors and reviewers,

We are very grateful for your constructive comments and suggestions for our manuscripts entitled “Effects of high-intensity interval training versus moderate-intensity continuous training on cardiorespiratory and exercise capacity in patients with coronary artery disease: a systematic review and meta-analysis”(ID:PONE-D-24-35383).Your comments are very valuable and helpful for improving our manuscripts.in the following,the responses to all the comment are provided one by one.

We have tried our best to make all the revisions clear,and we hope that the revised manuscript can satisfy the requirements for publication.

The main revisions in the new manuscript are:

**Reply to the editors suggestions for article modification**

1. This study was modified according to the format template for viewing PLOS ONE, which meets the format requirements of PLOS ONE.
2. We supplemented our research with the help of funders: We are very grateful to the Chengdu Fourth People's Hospital for supporting our work, especially funders Wu Dongmei and Yue Yuchuan for their help in research design, financial support and manuscript preparation.

We have added the relevant information of Financial Disclosure:

Project number: XJLL2023007;

Funded by: The Fourth People's Hospital of Chengdu;

Funder: Dong mei Wu(Funders of this study);Yue Yuchuan (Funders of this study).

3. We have described in the manuscript a table of the authors, years and other necessary contents of the included documents in this study, as well as the reasons for the excluded documents. All the included and excluded documents do not include unpublished studies.
4. We have uploaded a risk and quality assessment table of literature bias conducted in this study, which includes the reasons for inclusion, in a supplementary document3,5.

**Reply to the reviewer's suggestions for article modification**

**Reply to the reviewer 1**

1. The content of treadmill and Cycle ergometer you mentioned has been deleted in the abstract.See page 2-3 for details.
2. According to your suggestion that the P-value of various parameters of significance is >0.05, we modified the corresponding expression in the article by consulting the relevant system evaluation of HIIT and MICT and based on the data analysis results of this study.Has been marked in the manuscript.
3. About the content of the flow chart you mentioned. We have revised and updated the flowchart content.See Fig 1 for details.

**Reply to the reviewer 2**

1. According to your definition of PeakVO2 in keywords, we have provided the full name in the keyword section.
2. Regarding the email address(1903222804@qq.com) you mentioned, I have consulted the corresponding author, and this is the only email address she used in the submission process.
3. In the preface you mentioned, we have simplified the epidemiology of cardiovascular diseases in the preface, in order to highlight the benefits of exercise on exercise ability of cardiovascular diseases and introduce the focus of this study.See page 3-5 for details.
4. According to your suggestion that HIIT and MICT should take into account the effects of different exercise intensity, duration, and frequency on PeakVO2, we sincerely appreciate your input. In response, we have extended the duration of each exercise session and increased the weekly frequency of both HIIT and MICT for subgroup analysis. You can find detailed information in the Fig8(c d) provided on the article page. However, due to variations in calculation/evaluation methods used for exercises with different intensities (such as HRmax, HRpeak, VO2max, and Workload), this study did not conduct subgroup analysis based on exercise intensity. Additionally, some evaluation methods had a varying number of indicators. For instance, only one study utilized VO2max as a measure of training intensity. Combining or conducting subgroup analysis using different calculation/evaluation methods could introduce heterogeneity in results or bias towards subgroups with more literature available. As we did not perform this specific subgroup analysis mentioned above, we have acknowledged this limitation in our explanation.
5. According to your PRISMA standard suggestions, we have added the specific details of each process in PRISMA, such as adding the location of images and supplementary files.
6. According to your suggestion, by referring to similar systematic evaluation and combining with the analysis of the results of this study, we have decided to include the pictures with publication bias in the attachment, and this decision will not increase the difficulty of understanding the full text.
7. According to your suggestions, this study has re-searched the following databases (Pubmed, Web of Science, Embase, Cochrane Library, CLNAHL) using the specified search terms. Based on the input from researchers Gao and Zhang, we included another researcher Zhu to collaborate in literature screening. During the screening process, we encountered a disagreement and sought advice from a fourth researcher Yue. Throughout the screening process, we retained and labeled documents that were included in the initial search while excluding others. We also compared documents from the first search with those from the second search. As of September 26th, a total of 1432 literatures were searched; however, no updated literature relevant to this systematic evaluation was found. The flow chart has been updated accordingly and Boolean operation symbols have been added to our search files. No filters were applied during retrieval.
8. Based on your suggestions, we added a more cautious interpretation of the results and discussion by referring to other studies and reviewing the discussion.

Sincerely,

Corresponding author.
